# Retraction: Overexpressed miR‐335‐5p Reduces Atherosclerotic Vulnerable Plaque Formation in Acute Coronary Syndrome

**DOI:** 10.1002/jcla.70093

**Published:** 2025-08-13

## Abstract

RETRACTION: SunD. MaT. ZhangY. ZhangF. CuiB. Overexpressed miR‐335‐5p Reduces Atherosclerotic Vulnerable Plaque Formation in Acute Coronary Syndrome Journal of Clinical Laboratory Analysis 35, no. 2 (2021): e23608 10.1002/jcla.23608 33277957 PMC7891542

The above article, published online on 5 December 2020 in Wiley Online Library (http://onlinelibrary.wiley.com/), has been retracted by agreement between the authors; the journal Editor‐in‐Chief, Rong Fu; and Wiley Periodicals, LLC. The authors notified the journal of potential data irregularities. An investigation by the publisher confirmed that elements in Figure 7 were duplicated, and that elements of Figure 9 were duplicated from a previously published article by a different author group (Zhang et al. 2020 [https://www.frontiersin.org/journals/physiology/articles/10.3389/fphys.2020.00804/full]). The retraction has been agreed due to the evidence of image duplication and manipulation which fundamentally compromises the conclusions presented in the article. The authors agree with this decision.

RETRACTION: Sun D. Ma T. Zhang Y. Zhang F. Cui B. Overexpressed miR‐335‐5p Reduces Atherosclerotic Vulnerable Plaque Formation in Acute Coronary Syndrome Journal of Clinical Laboratory Analysis 35, no. 2 (2021): e23608 10.1002/jcla.23608 33277957 PMC7891542

The above article, published online on 5 December 2020 in Wiley Online Library (http://onlinelibrary.wiley.com/), has been retracted by agreement between the authors; the journal Editor‐in‐Chief, Rong Fu; and Wiley Periodicals, LLC. The authors notified the journal of potential data irregularities. An investigation by the publisher confirmed that elements in Figure 7 were duplicated, and that elements of Figure 9 were duplicated from a previously published article by a different author group (Zhang et al. 2020 [https://www.frontiersin.org/journals/physiology/articles/10.3389/fphys.2020.00804/full]). The retraction has been agreed due to the evidence of image duplication and manipulation which fundamentally compromises the conclusions presented in the article. The authors agree with this decision.

